# Supplementary material for: The causal role of circulating inflammatory markers in osteoporosis: a bidirectional Mendelian randomized study
Source: Front Immunol. 2024 Jul 18;15:1412298. doi: 10.3389/fimmu.2024.1412298 (PMC11291241; doi:10.3389/fimmu.2024.1412298)
Supplement: Supplementary file 1 [file DataSheet_1.zip › supplementary material/CIM-OSTEOPOROSIS /GCST90274794.csv_forest.pdf]

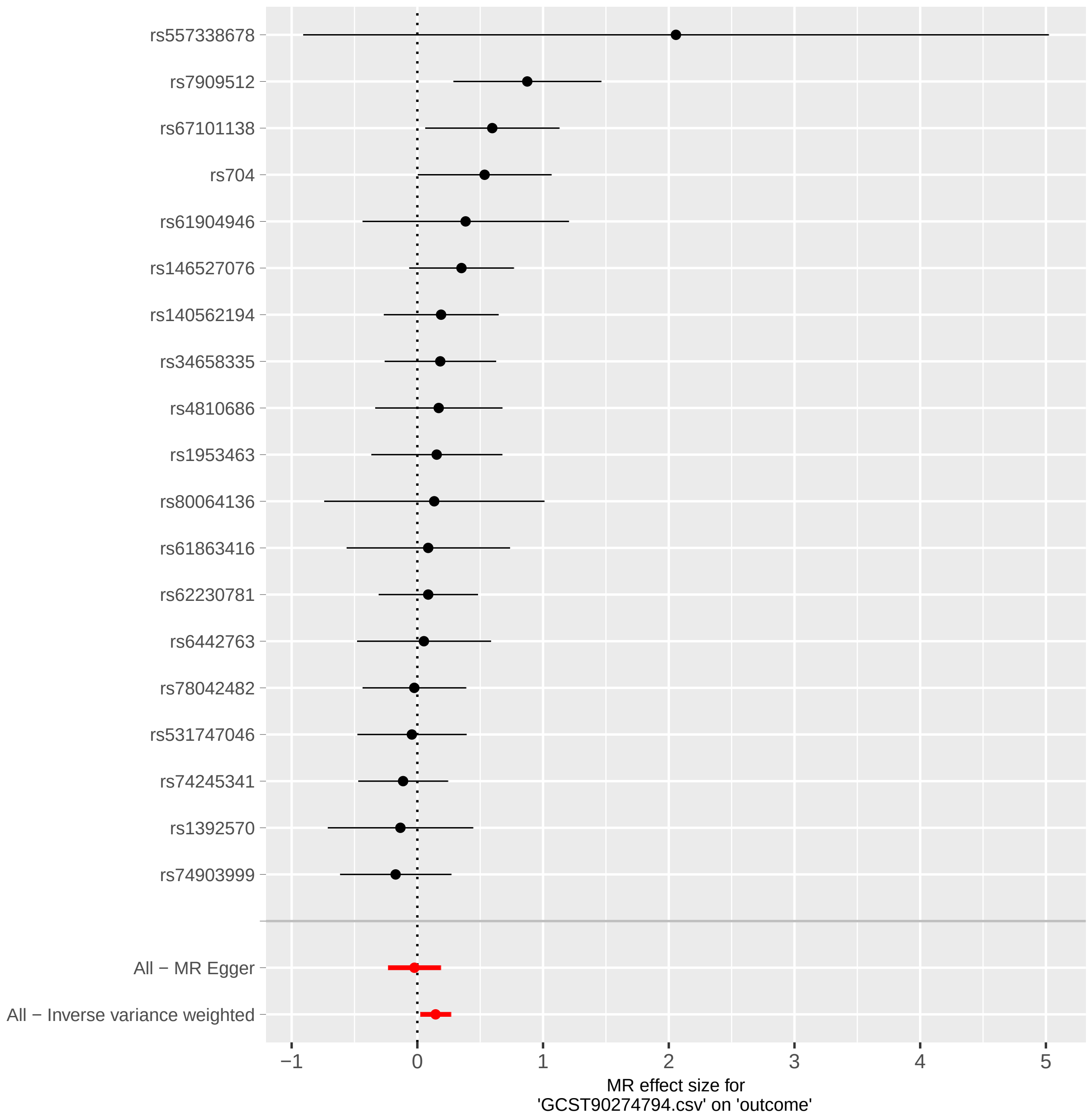

rs557338678
rs7909512
rs67101138
rs704
rs61904946
rs146527076
rs140562194
rs34658335
rs4810686
rs1953463
rs80064136
rs61863416
rs62230781
rs6442763
rs78042482
rs531747046
rs74245341
rs1392570
rs74903999
All − MR Egger
All − Inverse variance weighted
−1
0
1
2
3
4
5
MR effect size for
'GCST90274794.csv' on 'outcome'
